# On the embedding of convex spaces in stratified *L*-convex spaces

**DOI:** 10.1186/s40064-016-3255-5

**Published:** 2016-09-20

**Authors:** Qiu Jin, Lingqiang Li

**Affiliations:** Department of Mathematics, Liaocheng University, Hunan Road, Liaocheng, 252059 People’s Republic of China

**Keywords:** Fuzzy set, Convex space, Stratified *L*-convex space, Embedding functor, Subcategory

## Abstract

Consider *L* being a continuous lattice, two functors from the category of convex spaces (denoted by **CS**) to the category of stratified *L*-convex spaces (denoted by *SL*-**CS**) are defined. The first functor enables us to prove that the category **CS** can be embedded in the category *SL*-**CS** as a reflective subcategory. The second functor enables us to prove that the category **CS** can be embedded in the category *SL*-**CS** as a coreflective subcategory when *L* satisfying a multiplicative condition. By comparing the two functors and the well known Lowen functor (between topological spaces and stratified *L*-topological spaces), we exhibit the difference between (stratified *L*-)topological spaces and (stratified *L*-)convex spaces.

## Background

Abstract convexity theory is an important branch of mathematics (Van De Vel 1993). The notion of convexity considered here is considerably broader the classical one; specially, it is not restricted to the context of vector spaces. Now, a convexity on a set is a family of subsets closed for intersection and directed union. Convexities exist in many different mathematical research areas, such as convexities in lattices (Van De Vel 1984; Varlet 1975), convexities in metric spaces and graphs (Lassak 1977; Menger 1928; Soltan 1983) and convexities in topology (Chepoi 1994; Eckhoff 1968; Sierkama 1975; Van Mill 1977).

With the development of fuzzy mathematics, convexity has been interrelated to fuzzy set theory. Many authors have investigated fuzzy convexity by taking the interval [0, 1] as the truth table (Philip 2010; Rosa 1994a, b). However, as (Goguen 1967) pointed out, in some situations it may be impossible to represent degrees of membership by the linearly ordered set [0, 1]. Thus some lattice structures were proposed to replace the interval [0, 1] as the truth value table for membership degrees.

It is easily seen that there is some similarity between convexity and topology (a family of subsets of a set closed for union and finite intersection). Thus, similar to lattice-valued topologies (Höhle and Rodabaugh 1999; Li and Li 2015; Liu and Luo 1997; Sostak 1985; Wang 1988; Ying 1991), there are at least three kinds of lattice-valued convexities, namely, *L*-convexity (Maruyama 2009; Pang and Shi 2016), *M*-fuzzifying convexity (Shi and Xiu 2014; Shi and Li 2016) and (*L*, *M*)-convexity (Xiu 2015), where *L* and *M* are some complete lattices. When $L=2$, an (*L*, *M*)-convexity is precisely an *M*-fuzzifying convexity; when $M=2$, an (*L*, *M*)-convexity is precisely an *L*-convexity; and when $L=M=2$, an (*L*, *M*)-convexity is precisely a convexity. Similar to (lattice-valued) topology, the categorical relationships between convexity and latticed-valued convexity is an important direction of research. When *L* being a completely distributive complete lattices with some additional conditions, Pang and Shi (2016) proved that the category of convex spaces can be embedded in the category of stratified *L*-convex spaces as a coreflective subcategory.

In this paper, we shall continue to study the categorical relationships between convex spaces and stratified *L*-convex spaces. We shall investigate two embedding functors from the category of convex spaces (denoted by **CS**) to the category of stratified *L*-convex spaces (denoted by *SL*-**CS**). The first functor enables us to prove that the category **CS** can be embedded in the category *SL*-**CS** as a reflective subcategory when *L* being a continuous lattice. The second functor enables us to prove that the category **CS** can be embedded in the category *SL*-**CS** as a coreflective subcategory when the continuous lattice *L* satisfying a multiplicative condition. The second functor is an extension of Pang and Shi’s functor (2016) from the lattice-context. Precisely, from completely distributive complete lattice to continuous lattice. And the second functor can be regarded as an analogizing of the well known (extended) Lowen functor between the category of topological spaces and the category of stratified *L*-topological spaces (Höle and Kubiak 2007; Lai and Zhang 2005; Li and Jin 2011; Lowen 1976; Warner 1990; Yue and Fang 2005). By comparing the two functors and Lowen functor, we exhibit the difference between (stratified *L*-)topological spaces and (stratified *L*-)convex spaces from the categorical sense.

The contents are arranged as follows. In “Preliminaries” section, we recall some basic notions as preliminary. In “**CS** reflectively embedding in *SL*-**CS**” section, we present the reflective embedding of the category **CS** in the category *SL*-**CS**. In “**CS** coreflectively embedding in *SL*-**CS**” section, we focus on the coreflective embedding of the category **CS** in the category *SL*-**CS**. Finally, we end this paper with a summary of conclusion.

## Preliminaries

Let $L=(L,\le ,\vee ,\wedge ,0,1)$ be a complete lattice with 0 is the smallest element, 1 is the largest element. For $a,b\in L$, we say that *a* is way below *b* (in symbol, $a\ll b$) if for all directed subsets $D\subseteq L$, $b \le \vee D$ always implies that $a\le d$ for some $d \in D$. A complete lattice *L* is said to be continuous if $\forall x\in L$, $x = \vee \Downarrow x$, where $\Downarrow x=\{y\in L|\ y\ll x\}$ (Gierz et al. 2003). For a directed subset $D\subseteq L$, we use $\vee ^\uparrow D$ to denote its union.

Throughout this paper, *L* denote a continuous lattice, unless otherwise stated. The continuous lattice has a strong flavor of theoretical computer science (Gierz et al. 2003). The following lemmas collect some properties of way below relation on a continuous lattice.

### **Lemma 1**

(Gierz et al. 2003) *(1)*$$a\ll b\Rightarrow a\le b$$, *(2)*$$a\le b\ll c\le d$$$$\Rightarrow a\ll d$$, *(3)*$$a\ll b \Rightarrow \exists c$$*such that*$$a\ll c\ll b$$, *(4)*$$a\ll \vee ^\uparrow D\Rightarrow a\ll d$$*for some*$$d\in D$$.

### **Lemma 2**

(Gierz et al. 2003) *LetLbe a continuous lattice and let*$\{a_{j,k}|\ j\in J, k\in K(j)\}$*be a nonempty family of element inLsuch that*$\{a_{j,k}|\ k\in K(j)\}$*is directed for all*$j\in J$. *Then the following identity holds.*

$$\begin{aligned} {\mathbf{(DD)}}\ \ \ \ \ \ \ \ \ \bigwedge _{j\in J}{\mathop \bigvee \limits _{k\in K(j)}}^\uparrow a_{j,k}={\mathop \bigvee \limits _{h\in N}}^\uparrow \bigwedge _{j\in J}a_{j,h(j)}, \end{aligned}$$

*whereNis the set of all choice functions*$h:J\longrightarrow \bigcup _{j\in J}K(j)$*with*$h(j)\in K(j)$*for all*$j\in J$.

Let *X* be a nonempty set, the functions $X\longrightarrow L$, denoted as $L^X$, are called the *L*-subsets on *X*. The operators on *L* can be translated onto $L^X$ in a pointwise way. We make no difference between a constant function and its value since no confusion will arise. For a crisp subset $A\subseteq X$, we also make no difference between *A* and its characteristic function $\chi _A$. Clearly, $\chi _A$ can be regarded as an *L*-subset on *X*. Let $f:X\longrightarrow Y$ be a function. Then define $f^{\leftarrow }_L: L^Y\longrightarrow L^X$ by $f^{\leftarrow }_L(\lambda )=\lambda \circ f$ for $\lambda \in L^Y$. For a nonempty set *X*, let $2^X$ denotes the powerset of *X*.

### **Definition 1**

(Van De Vel 1993) A subset ${\mathcal {C}}$ of $2^X$ is called a convex structure on *X* if it satisfies:

1. $$\emptyset ,X\in {\mathcal {C}}$$;
2. if $\{A_j\}_{j\in J}\subseteq {\mathcal {C}}$, then $\bigcap _{j\in J}A_j\in {\mathcal {C}}$, where $J\ne \emptyset$;
3. if $\{A_j\}_{j\in J}\subseteq {\mathcal {C}}$ is directed, then its union, denoted as $\bigcup _{j\in J}^\uparrow A_j$, belongs to ${\mathcal {C}}$.

The pair $(X,{\mathcal {C}})$ is called a convex space. A mapping $f:(X,{\mathcal {C}}_X)\longrightarrow (Y,{\mathcal {C}}_Y)$ is called convexity-preserving (CP, in short) provided that $B \in {\mathcal {C}}_Y$ implies $f^{-1} (B)\in {\mathcal {C}}_X$. The category whose objects are convex spaces and whose morphisms are CP mappings will be denoted by **CS**.

### **Definition 2**

(Maruyama 2009; Pang and Shi 2016) A subset ${\mathfrak {C}}$ of $L^X$ is called an *L*-convex structure on *X* if it satisfies:

1. $$0,1\in {\mathfrak {C}}$$;
2. if $\{\lambda _j\}_{j\in J}\subseteq {\mathfrak {C}}$, then $\bigwedge _{j\in J} \lambda _j \in {\mathfrak {C}}$, where $J\ne \emptyset$;
3. if $\{\lambda _j\}_{j\in J}\subseteq {\mathfrak {C}}$ is directed, then its union, denoted as $\bigvee _{j\in J}^\uparrow \lambda _j$, belongs to ${\mathfrak {C}}$.

The pair $(X,{\mathfrak {C}})$ is called an *L*-convex space and it is called stratified if it satisfies moreover (LCS): $\forall \alpha \in L, \alpha \in {\mathfrak {C}}$.

A mapping $f:(X,{\mathfrak {C}}_X)\longrightarrow (Y,{\mathfrak {C}}_Y)$ between stratified *L*-convex spaces is called *L*-convexity-preserving (*L*-CP, in short) provided that $\mu \in {\mathfrak {C}}_Y$ implies $f^\leftarrow (\mu )\in {\mathfrak {C}}_X$. The category whose objects are stratified *L*-convex spaces and whose morphisms are *L*-CP mappings will be denoted by *SL*-**CS**.

Finally, we recall some categorical notions from Adámek et al. (1990).

### **Definition 3**

(Adámek et al. 1990) Suppose that **A** and **B** are concrete categories; $$F: {\mathbf{A}}\longrightarrow {\mathbf{B}}$$ and $$G: {\mathbf{B}}\longrightarrow {\mathbf{A}}$$ are concrete functors. The pair (*F*, *G*) is called a *Galois correspondence* if $$F\circ G\le {\mathrm{id}}$$ in the sense that for each $$Y\in {\mathbf{B}}$$, $${\mathrm{id}}_Y: F\circ G(Y)\longrightarrow Y$$ is a **B**-morphism; and $${\mathrm{id}}\le G\circ F$$ in the sense that for each $$X\in {\mathbf{A}},$$$${\mathrm{id}}_X: X\longrightarrow G\circ F(X)$$ is an **A**-morphism.

If (*F*, *G*) is a Galois correspondence, then *F* is a left adjoint of *G* (equivalently, *G* is a right adjoint of *F*), hence *F* and *G* form an adjunction $F\vdash G:{\mathbf{A}}\rightharpoonup {\mathbf{B}}$.

## **CS** reflectively embedding in *SL*-**CS**

In this section, we shall present a functor from the category **CS** to the category *SL*-**CS**, and then by using it to prove that the category **CS** can be embedded in the category *SL*-**CS** as a reflective subcategory.

At first, we fix some notations. For $a\in L$, $x\in X$, we denote $x_a$ as the *L*-subset values *a* at *x* and values 0 otherwise. For $\lambda \in L^X$, let $pt(\lambda )=\{x_a|\ a\ll \lambda (x)\}$ and let $Fin(\lambda )$ denote the set of finite subset of $pt(\lambda )$. Obviously, $\lambda =\vee pt(\lambda )=\vee \{\vee F|\ F\in Fin(\lambda )\}$.

### **Definition 4**

Let $(X,{\mathcal {C}})$ be a convex space and let ${\mathfrak {B}}=\{a\wedge U|\ a\in L,U\in {\mathcal {C}}\}.$ Then the set $\omega _L^1({\mathcal {C}})$ defined below is a stratified *L*-convex structure on *X*,

$$\begin{aligned} \omega _L^1({\mathcal {C}})=\left\{ {\mathop \bigvee \limits _{j\in J}}^\uparrow \mu _j|\ \{\mu _j\}_{j\in J} \subseteq {\mathfrak {B}}\ {\mathrm{is \ directed}}\right\} . \end{aligned}$$

### *Proof*

(LCS). It is obvious.

(LC2). It is easily seen that ${\mathfrak {B}}$ is closed for the operator $\wedge$.

For any $\{\lambda _j\}_{j\in J}\subseteq \omega _L^1({\mathcal {C}})$, let $\lambda _j={\mathop \bigvee \nolimits _{k\in K(j)}}^\uparrow \mu _{j,k}$. Then

$$\begin{aligned} \bigwedge _{j\in J}\lambda _j=\bigwedge _{j\in J}{\mathop \bigvee \limits _{k\in K(j)}}^\uparrow \mu _{j,k}\mathop {=}\limits ^{\mathbf{(DD)}}{\mathop \bigvee \limits _{h\in N}}^\uparrow \bigwedge _{j\in J}\mu _{j,h(j)}\in \omega _L^1({\mathcal {C}}), \end{aligned}$$

where *N* is the set of all choice functions $h:J\longrightarrow \bigcup _{j\in J}K(j)$ with $h(j)\in K(j)$ for all $j\in J$.

(LC3) Let $\{\lambda _j\}_{j\in J}\subseteq \omega _L^1({\mathcal {C}})$ be directed and $\lambda _j={\bigvee }^\uparrow _{k\in K(j)}\mu _{j,k}$. We denote

$$\begin{aligned} \lambda :={\mathop \bigvee \limits _{j\in J}}^\uparrow \lambda _j={\mathop \bigvee \limits _{j\in J}}^\uparrow {\mathop \bigvee \limits _{k\in K(j)}}^\uparrow \mu _{j,k}. \end{aligned}$$

Let $\sigma :Fin(\lambda )\longrightarrow {\mathfrak {B}}$ be a function defined by

$$\begin{aligned} \sigma (F)=\wedge \{\mu _{j,k}|\ \vee F\le \mu _{j,k}\}. \end{aligned}$$

1. $$\sigma$$ is definable.

Let $F\in Fin(\lambda )$. Then For each $x_a\in F$, we have $a\ll \lambda (x)={\mathop \bigvee \nolimits _{j\in J}}^\uparrow \lambda _j(x)$. It follows by Lemma 1 (4) that $a\ll \lambda _{j_{x_a}}(x)$ for some $j_{x_\alpha }\in J$. Since $\{\lambda _j\}_{j\in J}$ is directed then there exists a $j\in J$, denote as $j_F$, such that $\lambda _{j_{x_a}}\le \lambda _{j_F}$ for all $j_{{x_a}}$. By Lemma 1 (2) we get $a\ll \lambda _{j_F}(x)$. This shows that $F\in Fin(\lambda _{j_F})$. By a similar discussion on $\lambda _{j_F}$ we have that $F\in Fin(\mu _{j_F,k_F})$ for some $k_{F}\in K(j_{F})$. It follows that $\vee F\le \mu _{j_F,k_F}$.

We have proved that for any $F\in Fin(\lambda )$, there exists a $\mu _{j,k}$ such that $\vee F\le \mu _{j,k}$. Because ${\mathfrak {B}}$ is closed for $\wedge$, we get that $\sigma$ is definable.

2. The set $\{\sigma (F)|\ F\in Fin(\lambda )\}$ is directed.

It is easily seen that $\sigma$ is order-preserving. That is, if $F_1, F_2\in Fin(\lambda )$ and $F_1\subseteq F_2$ then $\sigma (F_1)\le \sigma (F_2)$. Thus for any $F_1,F_2\in Fin(\lambda )$, it follows that $F_1\cup F_2\in Fin(\lambda )$ and $\sigma (F_1), \sigma (F_2)\le \sigma (F_1\cup F_2)$. This shows that $\{\sigma (F)|\ F\in Fin(\lambda )\}$ is directed.

3. $$\lambda =\bigvee \{\sigma (F)|\ F\in Fin(\lambda )\}$$.

For any $F\in Fin(\lambda )$, it is easily observed that $\vee F\le \sigma (F)\le \lambda .$ Then it follows that

$$\begin{aligned} \lambda =\vee \{\vee F|\ F\in Fin(\lambda )\}\le \vee \{\sigma (F)|\ F\in Fin(\lambda )\}\le \lambda . \end{aligned}$$

This means that $\lambda =\bigvee \{\sigma (F)|\ F\in Fin(\lambda )\}$.

By a combination of (1)–(3), we have proved that $\lambda \in \omega _L^1({\mathcal {C}})$. $\square$

### **Lemma 3**

*Let *$(X,{\mathcal {C}})$*be a convex space. Then *$\chi _U\in \omega _L^1({\mathcal {C}})$*iff*$U\in {\mathcal {C}}$.

### *Proof*

The sufficiency is obvious. We check the necessity. Let $\chi _U\in \omega _L^1({\mathcal {C}})$. Then

$$\begin{aligned} \chi _U={\bigvee_{j\in J}}^\uparrow(a_j\wedge {U_j}) \end{aligned}$$

with $\forall j\in J, a_j\in L, U_j\in {\mathcal {C}}$ and $\{a_j\wedge {U_j}\}_{j\in J}$ is directed. Without loss of generality, we assume that $a_j\ne 0$ for all $j\in J$. It is easily seen that $\{U_j\}_{j\in J}$ is directed. In the following we check that

$$\begin{aligned} U={\bigcup_{j\in J}}^\uparrow U_j\in {\mathcal {C}}. \end{aligned}$$

On one hand, it is obvious that $U\supseteq U_j$ for any $j\in J$ and so $U\supseteq {\bigcup }^\uparrow _{j\in J}U_j$. On the other hand, for any $x\in U$ we have

$$\begin{aligned} {\bigvee_{j\in J}}^\uparrow (a_j\wedge {U_j})(x)=1, \end{aligned}$$

which means $x\in U_j$ for some $j\in J$. Thus $U\subseteq {\bigcup }^\uparrow _{j\in J}U_j$ as desired.

### **Proposition 1**

$f:(X,{\mathcal {C}}_X)\longrightarrow (Y,{\mathcal {C}}_Y)$*is CP iff*$f:(X,\mathcal \omega _L^1({C}_X))\longrightarrow (Y,\omega _L^1({\mathcal {C}}_Y))$*isL*-*CP.*

### *Proof*

Let $f:(X,{\mathcal {C}}_X)\longrightarrow (Y,{\mathcal {C}}_Y)$ be CP. Then for any $\lambda ={\bigvee }^\uparrow _{j\in J}(a_j\wedge {U_j})\in \omega _L^1({\mathcal {C}}_Y)$, we have

$$\begin{aligned} f^\leftarrow (\lambda )& = f^\leftarrow \left( {\bigvee }^\uparrow _{j\in J}(a_j\wedge {U_j})\right) ={\bigvee }^\uparrow _{j\in J}\left( a_j\wedge {f^{-1}(U_j)}\right) \in \omega _L^1({\mathcal {C}}_X). \end{aligned}$$

It follows that $f:(X,\mathcal \omega _L^1({C}_X))\longrightarrow (Y,\omega _L^1({\mathcal {C}}_Y))$ is *L*-CP. $\square$

Conversely, let $f:(X,\mathcal \omega _L^1({C}_X))\longrightarrow (Y,\omega _L^1({\mathcal {C}}_Y))$ be *L*-CP. Then for any $U\in {\mathcal {C}}_Y$, we have $\chi _U\in \omega _L^1({\mathcal {C}}_Y)$ and so ${f^{-1}(U)}=f^{\leftarrow }(\chi _ U)\in \omega _L^1({\mathcal {C}}_X)$. It follows by Lemma 3 that $f^{-1}(U)\in {\mathcal {C}}_X$. Thus $f:(X,{\mathcal {C}}_X)\longrightarrow (Y,{\mathcal {C}}_Y)$ is CP.

It is easily seen that the correspondence $(X,{\mathcal {C}})\mapsto (X,\omega _L^1({\mathcal {C}}))$ defines an embedding functor

$$\begin{aligned} \omega _L^1:{\mathbf{CS}}\longrightarrow L{\text{-}}{} {\mathbf{CS}}. \end{aligned}$$

### **Proposition 2**

(Pang and Shi 2016) *Let *$(X,{\mathfrak {C}})$*be a stratifiedL*-*convex space. Then the set*$\rho _L({\mathfrak {C}})=\{U\in 2^X|\ U\in {\mathfrak {C}}\}$*forms a convex structure onXand the correspondence*$(X,{\mathfrak {C}})\mapsto (X,\rho _L({\mathfrak {C}}))$*defines a concrete functor*

$$\begin{aligned} \rho _L:L{\text{-}} {\mathbf{CS}}\longrightarrow {\mathbf{CS}}. \end{aligned}$$

### **Theorem 1**

*The pair *$(\rho _L,\omega _L^1)$*is a Galois correspondence and*$\rho _L$*is a left inverse of*$\omega _L^1$.

### *Proof*

It is sufficient to show that $\rho _L\circ \omega _L^1({\mathcal {C}})={\mathcal {C}}$ for any $(X,{\mathcal {C}})\in$**CS** and $\omega _L^1\circ \rho _L({\mathfrak {C}})\subseteq {\mathfrak {C}}$ for any $(X,{\mathfrak {C}})\in L$-**CS**.

1. $\rho _L\circ \omega _L^1({\mathcal {C}})={\mathcal {C}}$. It follows immediately by Lemma 3.
2. $\omega _L^1\circ \rho _L({\mathfrak {C}})\subseteq {\mathfrak {C}}$. Let $\lambda \in \omega _L^1\circ \rho _L({\mathfrak {C}})$. Then $\lambda ={\bigvee }^\uparrow _{j\in J}(a_j\wedge {U_j})$ with $\forall j\in J, a_j\in L, {U_j}\in {\mathfrak {C}}$ and $\{a_j\wedge {U_j}\}_{j\in J}$ is directed. It follows by the definition of stratified *L*-convex space that $\lambda \in {\mathfrak {C}}$.

$$\square$$

### **Corollary 1**

*The category ***CS***can be embedded in the category SL*-**CS***as a reflective subcategory.*

## **CS** coreflectively embedding in *SL*-**CS**

In this section, we shall give a functor from the category **CS** to the category *SL*-**CS**, and then by using it to prove that the category **CS** can be embedded in the category *SL*-**CS** as a coreflective subcategory. This functor extends Pang and Shi’s functor (2016) from the lattice-context. Precisely, from completely distributive complete lattice to continuous lattice.

Firstly, we fix some notations used in this section.

Let $\lambda \in L^X$ and $a\in L$. Then the set $\lambda _{[a]}:=\{x\in X|\ a\le \lambda (x)\}$ and the set $\lambda _{(a)}:=\{x\in X|\ a\ll \lambda (x)\}$ are called the *a*-cut and strong *a*-cut of $\lambda$, respectively.

Let $a,b\in L$, we say that *a* is wedge below *b* (in symbol, $a\triangleleft b$) if for all subsets $D\subseteq L$, $y \le \vee D$ always implies that $x\le d$ for some $d \in D$. For each $a\in L$, denote $\beta (a)=\{b\in L|\ b\triangleleft a\}$.

The following lemma generalizes Huang and Shi’s result from lattice-context. Huang and Shi (2008) defined $\lambda _{(a)}:=\{x\in X|\ a\triangleleft \lambda (x)\}$ and assumed that *L* being completely distributive complete lattice.

### **Lemma 4**

*Let *$\lambda \in L^X$*and*$a\in L$. *Then* (1) $\lambda _{[a]}=\bigcap _{b\ll a}\lambda _{(b)}$; (2) $\lambda _{(a)}=\bigcup _{a\ll b}\lambda _{[b]}$.

### *Proof*

1. For any $b\ll a$, it follows by Lemma 1 (2) that $\lambda _{[a]}\subseteq \lambda _{(b)}$. Thus $\lambda _{[a]}\subseteq \bigcap _{b\ll a}\lambda _{(b)}$. Conversely, let $x\in \bigcap _{b\ll a}\lambda _{(b)}$. Then by Lemma 1 (1) we get $\forall b\ll a$, $\lambda (x)\ge b$, it follows that $\lambda (x)\ge \vee \Downarrow a=a$, i.e., $x\in \lambda _{[a]}$. Thus $\lambda _{[a]}\supseteq \bigcap _{b\ll a}\lambda _{(b)}$.
2. For any $a\ll b$, it follows by Lemma 1 (2) that $\lambda _{[b]}\subseteq \lambda _{(a)}$. Thus $\lambda _{(a)}\supseteq \bigcup _{a\ll b}\lambda _{[b]}$. Conversely, let $x\in \lambda _{(a)}$. Then by Lemma 1 (3) we have $b\in L$ such that $a\ll b\ll \lambda (x)$, it follows by Lemma 1 (1) that $\lambda (x)\ge b$, i.e., $x\in \lambda _{[b]}$. Thus $\lambda _{(a)}\subseteq \bigcup _{a\ll b}\lambda _{[b]}$.

### **Lemma 5**

*Let *$\{\lambda _j\}_{j\in J}\subseteq L^X$*be directed. Then*$(\bigvee ^{\uparrow }_{j\in J}\lambda _j)_{(a)}=\bigcup ^{\uparrow }_{j\in J}(\lambda _j)_{(a)}$*for any*$a\in L$.

### *Proof*

Let $x\in (\bigvee ^{\uparrow }_{j\in J}\lambda _j)_{(a)}$, i.e., $a\ll \bigvee ^{\uparrow }_{j\in J}\lambda _j(x)$. Then it follows immediately from Lemma 1 (4) that $a\ll \lambda _j(x)$, i.e., $x\in (\lambda _j)_{(a)}$ for some $j\in J$. This means $(\bigvee ^{\uparrow }_{j\in J}\lambda _j)_{(a)}\subseteq \bigcup ^{\uparrow }_{j\in J}(\lambda _j)_{(a)}$. The reverse inclusion holds obviously.

The way below relation $\ll$ on *L* is called multiplicative (Gierz et al. 2003) if $a\ll b, c$ implies $a\ll b\wedge c$.

### **Lemma 6**

*Assume that the way below relation *$\ll$*onLis multiplicative. Then for any*$\lambda \in L^X$*and any*$a\in L$, *the set*$\{\lambda _{(b)}|\ a\ll b, b\in L\}$*is directed if it is nonempty.*

### *Proof*

For any $\lambda _{(b)},\lambda _{(c)}$ with $a\ll b, c$, it follows by Lemma 1 (2) that $\lambda _{(b)},\lambda _{(c)}\subseteq \lambda _{(b\wedge c)}$. In addition, by the multiplicative condition we have $a\ll b\wedge c$. This proves that $\{\lambda _{(b)}|\ a\ll b, b\in L\}$ is directed.

Pang and Shi (2016) proved a similar result when *L* being a completely distributive complete lattice with the condition $\beta (a\wedge b)=\beta (a)\cap \beta (b)$ for any $a,b\in L$. It is easily seen that this condition is equivalent to that the wedge below relation on *L* is multiplicative. $\square$

### **Definition 5**

Let $(X,{\mathcal {C}})$ be a convex space and the way below relation $\ll$ on *L* be multiplicative. Then the set $\omega _L^2({\mathcal {C}})$ defined below is a stratified *L*-convex structure on *X*,

$$\begin{aligned} \omega _L^2({\mathcal {C}})=\left\{ \lambda \in L^X|\ \forall a\in L, \lambda _{[a]}\in {\mathcal {C}}\right\} . \end{aligned}$$

### *Proof*

The proofs of (LCS) and (LC2) are obvious. We only check (LC3) below.

Let $\{\lambda _j\}_{j\in J}\subseteq \omega _L^2({\mathcal {C}})$ be directed and $a\in L$. Then

$$\begin{aligned} \left( {\bigvee _{j\in J}}^\uparrow \lambda _j\right) _{[a]}&\mathop {=}\limits ^\mathrm{Lemma\, 4 (1)}&\bigcap _{b \in \Downarrow a}\left( {\bigvee _{j\in J}}^\uparrow \lambda _j\right) _{(b)}\mathop {=}\limits ^\mathrm{Lemma \,5}\bigcap _{b \in \Downarrow a}{\bigcup _{j\in J}}^\uparrow (\lambda _j)_{(b)}\\&\mathop {=}\limits ^{\mathrm{Lemma \,6,Lemma \,4 (2)}}&\bigcap _{b \in \Downarrow a}{\bigcup _{j\in J}}^\uparrow {\bigcup _{b\in \Downarrow c}}^\uparrow (\lambda _j)_{[c]}. \end{aligned}$$

It follows immediately that ${\bigvee _{j\in J}}^\uparrow \lambda _j\in \omega _L^2({\mathcal {C}})$ by $(\lambda _j)_{[c]}\in {\mathcal {C}}$ for any $j\in J, c\in L$ and *C* being closed for intersection and directed union. $\square$

Similar to Pang and Shi (2016), we can prove that the correspondence $(X,{\mathcal {C}})\mapsto (X,\omega _L^2({\mathcal {C}}))$ defines an embedding functor

$$\begin{aligned} \omega _L^2:{\mathbf{CS}}\longrightarrow L{\mathbf{{\text{-}}CS}}. \end{aligned}$$

Let $(X,{\mathfrak {C}})$ be a stratified *L*-convex space. Then Pang and Shi (2016) defined $\iota _L({\mathfrak {C}})$ as the finest convex structure on *X* which contains all $\lambda _{[a]}$ for all $\lambda \in {\mathfrak {C}}, a\in L$. They proved that the correspondence $(X,{\mathfrak {C}})\mapsto (X,\iota _L({\mathfrak {C}}))$ defined a concrete functor

$$\begin{aligned} \iota _L:L{\text{-}}{} {\mathbf{CS}}\longrightarrow {\mathbf{CS}}. \end{aligned}$$

Similar to Pang and Shi (2016), when the way below relation $\ll$ on *L* being multiplicative, we get the following results. $\square$

### **Theorem 2**

*The pair *$(\omega _L^2, \iota _L)$*is a Galois correspondence and*$\iota _L$*is a left inverse of *$\omega _L^2$.

### **Corollary 2**

*The category ***CS***can be embedded in the categorySL*-**CS***as a coreflective subcategory.*

### *Remark 1*

Let us replace the convex space $(X,{\mathcal {C}})$ in Definition 4 and Definition 5 with a topological space $(X,{\mathcal {T}})$. Then $\omega _L^2$ defines an embedding functor from the category of topological spaces to the category of stratified *L*-topological spaces. This functor was first proposed by Lowen (1976) for $L=[0,1]$ and then extended by many researchers (Höle and Kubiak 2007; Lai and Zhang 2005; Liu and Luo 1997; Wang 1988; Warner 1990). If we further remove the directed condition in $\omega _L^1$ then we also get an embedding functor from the category of topological spaces to the category of stratified *L*-topological spaces. By the definition of stratified *L*-topology, it is easily seen that $\omega _L^1({\mathcal {T}})\subseteq \omega _L^2({\mathcal {T}})$. Conversely, if $\lambda \in \omega _L^2({\mathcal {T}})$ then $\lambda =\bigvee _{a\in L}(a\wedge \lambda _{[a]})\in \omega _L^1({\mathcal {T}})$. Thus $\omega _L^1=\omega _L^2$ and it follows the following well known result. That is, the category of topological spaces can be embedded in the category of stratified *L*-topological spaces as a both reflective and coreflective subcategory.

### *Remark 2*

Does **CS** can be embedded in *L*-**CS** as a both reflective and coreflective subcategory? Now, we can not answer it. For a convex space $(X,{\mathcal {C}})$, the inclusion $\omega _L^1({\mathcal {C}})\subseteq \omega _L^2({\mathcal {C}})$ holds obviously. But the reverse inclusion seems do not hold. The reason is that for an *L*-subset $\lambda \in L^X$, the set $\{a\wedge \lambda _{[a]}|\ a\in L\}$ is generally not directed.

At last, we give two interesting examples to distinguish (*L*-)convex space from (*L*-) topological spaces.

### *Example 1*

An upper set *U* on *L* is called Scott open if for each directed set $D \subseteq L$, $\vee ^\uparrow D\in U$ implies that $d\in U$ for some $d\in D$. It is known that the Scott open sets on *L* form a topology *L*, called the Scott topology (Gierz et al. 2003). It is not difficult to check that the Scott open sets on *L* do not form a convex structure on *L* since they are not closed for intersection.

### *Example 2*

An *L*-filter (Höhle and Rodabaugh 1999) on a set *X* is a function ${\mathcal {F}}:L^X\longrightarrow L$ such that for all $\lambda ,\mu \in L^X$, (F1) ${\mathcal {F}}(0)=0$, ${\mathcal {F}}(1)=1$; (F2) $\lambda \le \mu \Rightarrow {\mathcal {F}}(\lambda )\le {\mathcal {F}}(\mu )$; (F3) ${\mathcal {F}}(\lambda )\wedge {\mathcal {F}}(\mu )\le {\mathcal {F}}(\lambda \wedge \mu )$.

The set of *L*-filters on *X* is denoted by ${\mathcal {F}}_L(X)$. Since ${\mathcal {F}}_L(X)$ is a subset of $L^{(L^X)}$, hence, there is a natural partial order on ${\mathcal {F}}_L(X)$ inherited from $L^{(L^X)}$. Precisely, for ${\mathcal {F}}, {\mathcal {G}}\in {\mathcal {F}}_L(X)$, ${\mathcal {F}}\le {\mathcal {G}}\Leftrightarrow \forall \lambda \in L^X,{\mathcal {F}}(\lambda )\le {\mathcal {G}}(\lambda ).$

It is known that ${\mathcal {F}}_L(X)$ is closed for intersection, but is not closed for union (Fang 2010; Jäger 2001). In the following, we check that ${\mathcal {F}}_L(X)$ is closed for directed union.

Let $\{{\mathcal {F}}_j\}_{j\in J}\subseteq {\mathcal {F}}_L(X)$ be directed. Then it is readily seen that $\bigvee ^\uparrow _{j\in J}{\mathcal {F}}_j$ satisfies the conditions (F1) and (F2). Taking $\lambda ,\mu \in L^X$, then

$$\begin{aligned}&\left( {\mathop \bigvee \limits _{j\in J}}^\uparrow {\mathcal {F}}_j\right) (\lambda )\wedge \left( {\mathop \bigvee \limits _{j\in J}}^\uparrow {\mathcal {F}}_j\right) (\mu )\mathop {=}\limits ^{\mathbf{(DD)}}{\mathop \bigvee \limits _{j,k\in J}}^\uparrow ({\mathcal {F}}_j(\lambda )\wedge {\mathcal {F}}_k(\mu )), \{{\mathcal {F}}_j\}_{j\in J}\ \mathrm{is\ directed}\\& \quad \le {\mathop \bigvee \limits _{j,k\in J}}^\uparrow ({\mathcal {F}}_{jk}(\lambda )\wedge {\mathcal {F}}_{jk}(\mu )), {\mathcal {F}}_j,{\mathcal {F}}_k\le {\mathcal {F}}_{jk},\ {\mathrm{(F3)}}\\&\quad \le {\mathop \bigvee \limits _{j,k\in J}}^\uparrow {\mathcal {F}}_{jk}(\lambda \wedge \mu )\le \left( {\mathop \bigvee \limits _{j\in J}}^\uparrow {\mathcal {F}}_{j}\right) (\lambda \wedge \mu ). \end{aligned}$$

Thus $\bigvee ^\uparrow _{j\in J}{\mathcal {F}}_j$ satisfies the condition (F3). We have proved that ${\mathcal {F}}_L(X)$ is closed for directed union.

1. Let $Y=L^X$ and ${\mathfrak {C}}=\{0,1\}\cup {\mathcal {F}}_L(X)$. Then it is easily seen that ${\mathfrak {C}}$ is an *L*-convex structure on *Y* but not an *L*-topology on *Y*.
2. If we call a function ${\mathcal {F}}:L^X\longrightarrow L$ satisfying (F2) and (F3) as a nearly *L*-filter on *X*. Let ${\mathcal {F}}_L^N(X)$ denote the set of nearly *L*-filters on *X*. Then it is easily seen that ${\mathcal {F}}_L^N(X)$ is a stratified *L*-convex structure on *Y* but not a stratified *L*-topology on *Y*.

Note that $L^X$ forms a continuous lattice. If replacing $L^X$ with a continuous lattice *M*, similar to (1)–(2), we can define (stratified) *L*-convex structure on *M*.

## Conclusions

When *L* being a continuous lattice, an embedding functor from the category **CS** to *SL*-**CS** is introduced, then it is used to prove that the category **CS** can be embedded in the category *SL*-**CS** as a reflective subcategory. When *L* being a continuous lattice with a multiplicative condition, Pang and Shi’s functor (2016) is generalized from the lattice context, then it is used to prove that the category **CS** can be embedded in the category *SL*-**CS** as a coreflective subcategory. It is well known that the category of topological spaces can be embedded in the category of stratified *L*-topological spaces as a both reflective and coreflective subcategory. But, we find that the category of convex spaces seem not be embedded in the category of stratified *L*-convex spaces as a both reflective and coreflective subcategory. This shows the difference between (stratified *L*-)topological spaces and (stratified *L*-) convex spaces from categorical sense.
